# Prevalence of non-communicable diseases and access to health care and medications among Yazidis and other minority groups displaced by ISIS into the Kurdistan Region of Iraq

**DOI:** 10.1186/s13031-017-0106-0

**Published:** 2017-04-06

**Authors:** Valeria Cetorelli, Gilbert Burnham, Nazar Shabila

**Affiliations:** 1 Center for Humanitarian Health, Johns Hopkins Bloomberg School of Public Health, Baltimore, MD USA; 2 Middle East Centre, London School of Economics and Political Science, London, UK; 3 Department of Community Medicine, College of Medicine, Hawler Medical University, Erbil, Kurdistan Region Iraq

**Keywords:** Iraq, Kurdistan Region, Displacement, Yazidis, Non-communicable diseases, Access to health care

## Abstract

**Background:**

The increasing caseload of non-communicable diseases (NCDs) in displaced populations poses new challenges for humanitarian agencies and host country governments in the provision of health care, diagnostics and medications. This study aimed to characterise the prevalence of NCDs and better understand issues related to accessing care among Yazidis and other minority groups displaced by ISIS and currently residing in camps in the Kurdistan Region of Iraq.

**Methods:**

The study covered 13 camps managed by the Kurdish Board of Relief and Humanitarian Affairs. A systematic random sample of 1300 households with a total of 8360 members were interviewed between November and December 2015. Respondents were asked whether any household members had been previously diagnosed by a health provider with one or more of four common NCDs: hypertension, diabetes, cardiovascular disease and musculoskeletal conditions. For each household member with an NCD diagnosis, access to health care and medications were queried.

**Results:**

Nearly one-third of households had at least one member who had been previously diagnosed with one or more of the four NCDs included in this study. Hypertension had the highest prevalence (19.4%; CI: 17.0–22.0), followed by musculoskeletal conditions (13.5%; CI: 11.4–15.8), diabetes (9.7%; CI: 8.0–11.7) and cardiovascular disease (6.3%; CI: 4.8–8.1). Individual NCD prevalence and multimorbidity increased significantly with age. Of those with an NCD diagnosis, 92.9% (CI: 88.9–95.5) had seen a health provider for this condition in the 3 months preceding the survey. In the majority of cases, care was sought from private clinics or hospitals rather than from the camp primary health care clinics. Despite the frequent access to health providers, 40.0% (CI: 34.4–46.0) were not taking prescribed medications, costs being the primary reason cited.

**Conclusion:**

New strategies are needed to strengthen health care provision for displaced persons with NCDs and ensure access to affordable medications.

## Background

Health care for displaced populations has traditionally focused on maternal and child care and treatment of communicable diseases [1]. While these traditional health priorities remain relevant, demographic and lifestyle changes are increasing the burden of non-communicable diseases (NCDs) in populations worldwide. This epidemiological shift poses new challenges for humanitarian agencies and host country governments. NCDs require more complex diagnostic and management capacity than many common communicable diseases. In the absence of regular care and access to medications, NCDs may result in complications requiring costly specialised care and have the potential to seriously compromise both quality of life and life expectancy [2, 3]. These required resources are often not available in health services for displaced populations.

During the summer of 2014, the so-called Islamic State of Iraq and Syria (ISIS) subjugated Nineveh governorate in Northern Iraq. Nineveh has historically been home to most of Iraq’s minority groups, including Yazidis, Assyrian and Chaldean Christians, Sabaean-Mandaeans, Turkmen, Shabak and Kaka’i. As ISIS militants seized cities, towns and villages across the governorate, they systematically attacked civilians based on religious identities, committing mass atrocities and forcing about one million people to flee their homes [4, 5]. Most members of targeted minorities sought refuge in the neighbouring Duhok governorate within the Kurdistan Region of Iraq, where they remain displaced in local communities or in camps.

To manage this large population influx, a Board of Relief and Humanitarian Affairs (BRHA) was established in January 2015 as a new governmental body within Duhok’s governmental structure [6]. By the end of the year, BRHA managed 13 camps hosting approximately 200,000 internally displaced persons (IDPs) from Nineveh governorate, and two further camps were under construction [7]. The vast majority of IDPs residing in BRHA camps are Yazidis. Originally from the area of Mount Sinjar in Nineveh governorate, the Yazidis have long been one of the most marginalised and impoverished communities in Iraq [8]. Considered “devil worshippers” by ISIS militants, they were singled out for particularly brutal treatment. Their displacement experience and limited pre-existing ties with local communities in Kurdistan have made them especially vulnerable and reliant on humanitarian assistance [5].

Ensuring access to adequate health services is a persisting concern in BRHA camps. Each camp is equipped with a Primary Health Care Clinic (PHCC) staffed with doctors and medical auxiliaries. These provide preventive and basic curative services under the management of Duhok’s Directorate of Health and partner NGOs. However, most PHCCs lack medical specialists and do not stock medications for NCDs [6, 7]. This study aims to characterise the prevalence of NCDs among the displaced population residing in BRHA camps and better understand issues of health care access. With the humanitarian crisis continuing, data from a population-based assessment can assist the Kurdistan Regional Government and partners provide efficient and effective care to persons with NCDs with limited resources.

## Methods

This study covered 13 BRHA camps –Bajed Kandala, Bardarash, Bersive, Chamisku, Dawdiya, Essian, Garmawa, Karbato, Khanke, Mamilian, Rwanga, Shariya and Sheikhan– hosting Yazidis and other groups displaced by the ISIS expansion in Nineveh governorate during the summer of 2014. Maps were available for all camps, and all shelters –tents or prefabricated caravans– within camps were numbered. A stratified systematic random sample of 100 households per camp was selected, yielding a total of 1300 households. For each camp, a sampling interval *k* was determined as the ratio of camp size to sample size. A random number from 1 to *k* was used to identified a starting household and every *k* ^th^ household was selected thereafter.

The field team consisted of four pairs of local Yazidi interviewers –one male and one female– and one survey supervisor. The interviewers were given a 3-day training session concerning the questionnaire, sampling methods, data collection using tablets, interview techniques and basic principles of human subjects’ protection. A pilot survey was conducted in October 2015 to finalise the questionnaire and provide the interviewers with the opportunity to practice interviews with the target population. Fieldwork took place in November and December 2015.

Interviews were conducted with the household head or a responsible adult in the household. Where possible, efforts were made to involve household adults other than the primary respondent in the interview if questions pertained to them, so as to improve the accuracy of response. The interviewers obtained verbal informed consent from all participants after reading a consent form outlining the purpose of the survey, its confidentiality and the voluntary nature of participation. If no suitable informant was present in the household, an attempt was made to revisit the household later on the same day. In case of non-contact or refusal to participate, the interviewers were instructed to conduct an interview with the household living in the next nearest tent or caravan. To protect the anonymity of respondents, no unique identifiers were recorded.

The questionnaire included a specific section on NCDs. Respondents were asked whether any household members, including both children and adults, had been previously diagnosed by a health provider with one or more of the four most common NCDs in the region: hypertension, diabetes, cardiovascular disease and musculoskeletal conditions [9–11]. The health provider’s diagnosis as reported by the person interviewed was recorded; no documentation was secured from a health facility to verify the diagnosed condition. For each household member reporting an NCD diagnosis, access to health care in the 3 months preceding the survey and use of prescribed medications were queried.

Data were collected with tablets using the Magpi platform (DataDyne LLC, Washington, DC). The analysis was performed using Stata 14 (College Station, Texas), adjusting for sampling design [12]. The prevalence of each of the four NCDs and the accompanying 95% confidence intervals were calculated at the household level and by age groups. Differences in prevalence were assessed using the chi-squared method. The data also allowed the prevalence of NCD multimorbidities to be identified. The percentage among those reporting an NCD diagnosis who had visited a health facility for the NCD condition in the 3 months preceding the survey and the percentage of those who were taking prescribed medications were computed. The odd ratios of taking medications by type of prescribing health facility were also estimated.

## Results

**Fig. 1:**
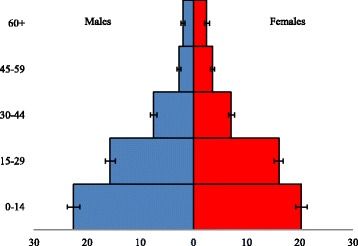
Population pyramid for IDPs in the surveyed households (with 95% CIs)

Of the 1300 selected households, 93 (7.2%) were replaced with households living in the next nearest shelter because a responsible adult was absent (6.5%) or refused to participate in the survey (0.7%). The interviewed households included a total of 8360 members (Table 1). All households had been displaced from their homes in Nineveh governorate during the ISIS attack, and 80.0% (CI: 77.9–81.9) were Yazidi. The mean household size was 6.9 (CI: 6.7–7.1). The proportion of males and females was 50.5% (CI: 49.5–51.5) and 49.5% (CI: 48.5–50.6) respectively; 42.9% (CI: 41.4–44.4) were children under 15 years of age (Fig. 1). There were 31.6% (CI: 28.7–34.6) of households which had at least one member who had been previously diagnosed with one or more of the four NCDs included in this study (Table 2). Hypertension was the most common (19.4%; CI: 17.0–22.0), followed by musculoskeletal conditions (13.5%; CI: 11.4–15.8), diabetes (9.7%; CI: 8.0–11.7) and cardiovascular disease (6.3%; CI: 4.8–8.1).

**Table 1:** Sample characteristics

	Unweighted N
Total individuals in surveyed households	8360
0–14	3670
15–29	2630
30–44	1193
45–59	550
60+	309
Individuals with one or more NCD diagnosis	460
Hypertension	228
Diabetes	137
Cardiovascular disease	76
Musculoskeletal conditions	184
Seen a health provider in the last 3 months	431
Camp PHCC	152
Hospital	128
Private clinic	151
Taken medications as prescribed	295

**Table 2:** Prevalence of NCDs and access to care (with 95% CIs)

	Hypertension % (CI)	Diabetes % (CI)	Cardiovascular disease % (CI)	Musculoskeletal conditions % (CI)
Households where any member(s) have condition	19.4 (17.0–22.0)	9.7 (8.0–11.7)	6.3 (4.8–8.1)	13.5 (11.4–15.8)
Prevalence by age-group				
0–14	0.0 (0.0–0.0)	0.0 (0.0–0.0)	0.1 (0.0–0.2)	0.1 (0.0–0.4)
15–29	0.2 (0.1–0.5)	0.2 (0.0–0.5)	0.0 (0.0–0.0)	0.6 (0.3–1.2)
30–44	4.4 (3.2–5.9)	1.3 (0.7–2.1)	1.3 (0.7–2.3)	4.2 (3.1–5.9)
45–59	23.9 (19.8–28.5)	12.8 (9.8–16.6)	5.1 (3.1–8.3)	12.3 (9.1–16.5)
60+	32.1 (26.3–38.4)	16.5 (12.2–21.8)	13.6 (9.4–19.3)	22.4 (16.6–29.6)
Seen a health provider in the last 3 months	97.1 (93.5–98.7)	97.9 (94.1–99.3)	94.7 (85.8–98.2)	88.3 (80.3–93.4)
Type of facility				
Camp PHCC	39.5 (33.0–46.4)	39.3 (29.9–49.7)	7.7 (2.7–20.0)	31.5 (23.5–40.7)
Public Hospital	29.1 (23.1–35.9)	29.5 (21.1–39.4)	38.5 (26.2–52.5)	24.8 (17.7–33.4)
Private Clinic	31.4 (25.2–39.3)	31.2 (22.6–41.4)	53.8 (39.8–67.2)	43.8 (34.8–53.2)
Taking medication as prescribed	68.5 (61.2–74.9)	67.2 (56.8–76.1)	59.1 (44.7–72.1)	34.8 (26.7–44.0)

**Fig. 2:**
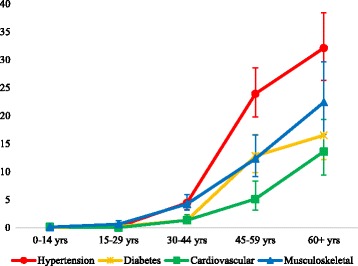
Prevalence of diagnosed NCDs by age-group (with 95% CIs)

None of the four NCD diagnoses were reported among children or young adults, but their prevalence increased with age among both men and women (*p* < 0.001). The most commonly diagnosed conditions among older adults were hypertension and musculoskeletal conditions. Hypertension prevalence rose from 4.4% (CI: 3.2–5.9) among those aged 30–44 to 23.9% (CI: 19.8–28.5) among those aged 45–59, and reached 32.1% (CI: 26.3–38.4) among those aged 60 and above. Similarly, prevalence of musculoskeletal conditions was 4.2% (CI: 3.1–5.9) among those aged 30–44, rising to 12.3% (CI: 9.1–16.5) among those aged 45–59 and to 22.4% (CI: 16.6–29.6) among those aged 60 and over (Fig. 2).

The prevalence of NCD multimorbidity was high, with 38.5% (CI: 33.0–44.2) of individuals having two or more conditions. The most common co-morbid conditions were hypertension and diabetes. The likelihood of having two or more NCDs increased significantly with age (*p* < 0.001): from 15.9% (CI: 9.6–25.3) among those aged 30–44, to 42.4% (CI: 33.8–51.4) among those aged 45–59 and 55.8% (CI: 45.7–65.5) among those aged 60 and over. There was no significant difference between men and women in the likelihood of reporting NCD multimorbidity (*p* = 0.339).

**Fig. 3:**
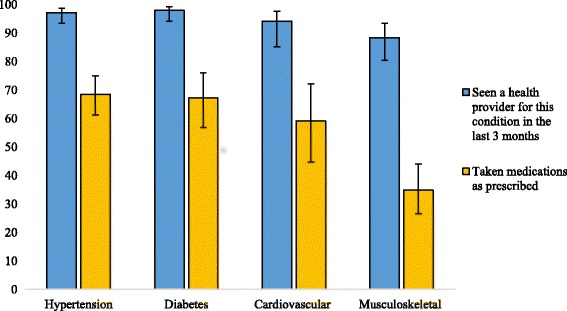
Access to NCD care and currently taking NCD medications (with 95% CIs)

The reported access to health care was high for all conditions. Among persons with hypertension 97.1% (CI: 93.5–98.7) had seen a health provider for this condition in the 3 months preceding the survey. For diabetes it was 97.9% (CI: 94.1–99.3), and for cardiovascular disease it was 94.7% (CI: 85.8–98.2). The frequency of having seen a health provider in the past 3 months was significantly less among those with musculoskeletal conditions (*p* < 0.001): 88.3% (CI: 80.3–93.4) (Fig. 3). For all four NCDs, the majority of patients sought care outside the camp, either in hospitals or private clinics. The number of visits to the camp PHCC in the past 3 months varied significantly by condition (*p* < 0.001), ranging from 39.5% (CI: 33.0–46.4) of persons with hypertension to 7.7% (CI: 2.7–20.0) of those with cardiovascular disease.

**Fig. 4:**
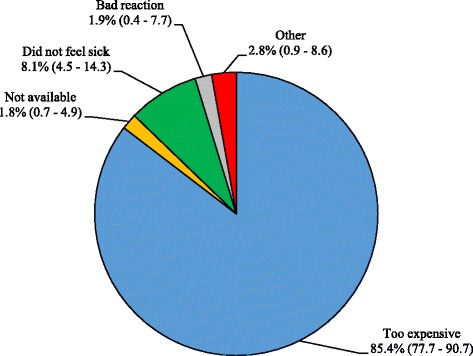
Reason for not taking prescribed NCD medications (with 95% CIs)

**Fig. 5:**
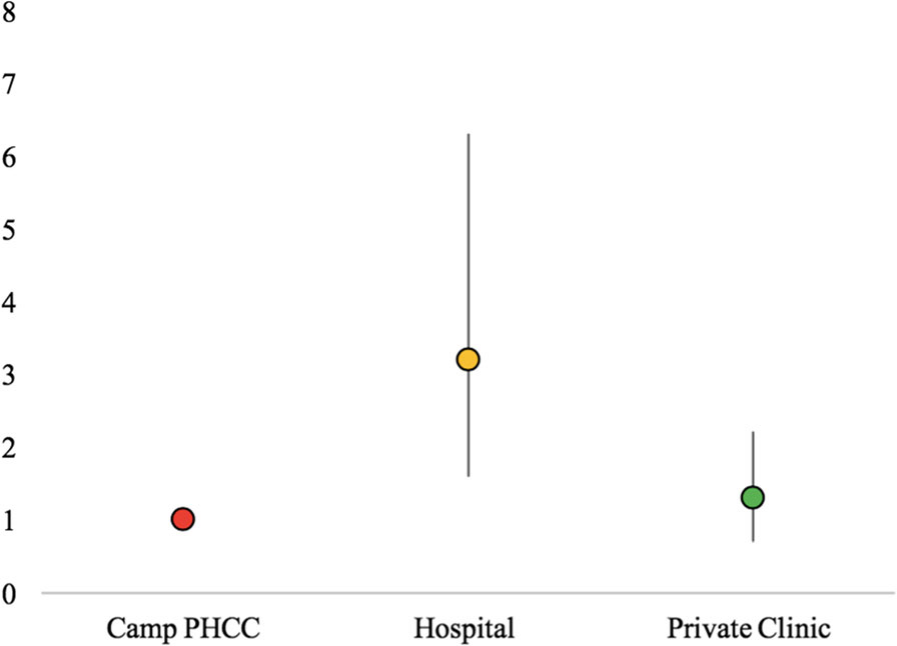
Odds ratios of taking medications as prescribed among those who had visited a hospital or private clinic compared to those who had visited the camp PHCC (with 95% CIs)

Having seen a health provider for their NCD in the past 3 months was considerably more frequent than currently taking prescribed medications for their condition (Fig. 3). The cost of medications was by far the major reason given by those who were not taking medications as prescribed by the health provider (Fig. 4). The adherence to prescribed treatment was 68.5% (CI: 61.2–74.9) for those with hypertension, 67.2% (CI: 56.8–76.1) for those with diabetes, 59.1% (CI: 44.7–72.1) for those with cardiovascular disease, and 34.8% (CI: 26.7–44.0) for those with musculoskeletal conditions (*p* < 0.001). Adherence to prescribed medications was associated with type of health facility visited (*p* < 0.001). Those who had sought care in hospital were 3.2 (CI: 1.6–6.3) times more likely to take medications as prescribed than those who had sought care in the camp PHCC (Fig. 5). There was no significant difference in terms of access to medications between those who had visited a private clinic and those who had sought care in the camp PHCC (*p* = 0.401).

## Discussion

The high prevalence of NCDs in displaced populations in the Middle East region constitutes an emerging challenge for humanitarian response [13]. New strategies for diagnosis and treatment need to be developed if excess morbidity and mortality from NCDs are to be prevented. In Iraq, as in most of the Middle East, NCD care is typically provided by specialists with little involvement of primary health care providers [14]. This is in contrast to other regions where the primary care physician provides much of the care for persons with uncomplicated NCDs. A fragmented care approach with higher service costs, can lead to reduced access by patients to health care providers and an inability to afford medications [15]. Building the competencies of primary care doctors to care for the common uncomplicated NCDs, establishing a referral system for medical complications, and ensuring common NCD medications are adequately stocked in PHCC pharmacies, which provide them free of charge, are steps that can be taken to improve care. Although the findings of this study are limited to the displaced Yazidis and other minorities in BRHA camps, the problems identified are common among the millions of displaced persons in the region.

After having experienced increasing persecutions in recent years, religious minorities in Iraq –especially the Yazidis– were subjected to an unprecedented brutality by ISIS during the summer of 2014 [4, 5]. Since then, they have remained displaced mainly in the Kurdistan Region of Iraq. A quick return home is unlikely, given the unpredictable and destructive nature of the continuing conflict. Even when territories are taken back from ISIS, the social fabric of these violence-torn communities cannot be easily reconstructed, and infrastructure is almost totally destroyed. This study provided a population-based assessment of prevalence and access to care and medications for NCDs among Yazidis and other groups currently residing in camps. With no end to this humanitarian crisis in sight, the study aim was to collect data that can inform longer-term planning to strengthen health care provision for those with NCDs in the Kurdistan Region.

The study showed a high burden of NCDs among Yazidis and other IDPs in BRHA camps. An estimated 19.4% (CI: 17.0–22.0) of households had at least one member who had been previously diagnosed with hypertension, 9.7% (CI: 8.0–11.7) had at least one member with diabetes, 6.3% (CI: 4.8–8.1) had at least one member with cardiovascular disease, and 13.5% (CI: 11.4–15.8) had at least one member with musculoskeletal conditions. These estimates are similar to those found by previous studies of displaced populations in the region. Surveys among Iraqi refugees in Jordan and Syria found prevalence of 19.6% (CI: 18.3–21.0) and 19.6% (CI: 18.0–21.3) for hypertension, 9.1% (CI: 8.2–10.1) and 7.6% (CI: 6.6–8.7) for diabetes, 6.7% (CI: 5.9–7.6) and 7.8% (CI: 6.8–9.0) for cardiovascular disease, and 18.5% (CI: 17.2–19.9) and 16.6% (CI: 15.1–18.2) for musculoskeletal conditions [10]. In recent surveys among Syrian refugees in Jordan and Lebanon, the estimated prevalence was respectively 26.3% (CI: 24.0–28.8) and 20.5% (CI: 18.2–23.0), 16.1% (CI: 14.4–18.0) and 9.9% (CI: 8.2–11.9), 12.3% (CI: 10.6–14.2) and 10.8% (CI: 9.3–12.6), 19.5% (CI: 17.3–21.9) and 21.2% (CI: 18.7–24.0) [16, 17]. The age patterns of NCDs are also aligned with previous studies, with older persons being disproportionately affected and much more likely to suffer from multimorbidities [18]. It is noteworthy that this and previous studies estimated prevalence based on self-reporting of a health provider’s diagnosis. This approach is likely to underestimate the true burden of NCDs in contexts where a potentially large number of cases remain undiagnosed. Another limitation of self-reporting is the possibility of misdiagnosis. A detailed assessment of health providers’ diagnosis and management of NCDs among conflict-affected populations and access to relevant medications would be an important next step in understanding the burden of NCDs on these populations.

Overall, 92.9% (CI: 88.9–95.5) of those reporting an NCD diagnosis had seen a health provider for their condition during the 3 months preceding the survey. This contrasts with the situation in central Iraq, where IDPs in informal settlements were reported to experience serious difficulties in accessing care [19]. The number of health facilities per population is higher in the Kurdistan Region than in other parts of Iraq [20], and the conflict-induced insecurity in Baghdad and Mosul has caused a migration of health providers to Kurdistan [21]. Better security in Kurdistan has also resulted in more assistance to IDPs from humanitarian agencies than in the rest of the country [22]. Data show that, in the majority of cases, care was sought outside camps, either in hospitals or private clinics. More qualitative research is needed on why many IDPs turn to the private sector, even when this implies a heavy financial burden on a household with limited resources, and why camp services remain underutilised. Significant differences in care-seeking by condition were identified. Only 7.7% (CI: 2.7–20.0) of those with cardiovascular disease had visited the camp PHCC, suggesting that services were perceived to be limited for this condition or that medications were not available. The lower rate of care-seeking among those with musculoskeletal conditions may be partly explained by reduced mobility making it difficult for them to travel to health facilities outside camps. Health services may have not been perceived as being helpful for these conditions.

Despite the relatively good access to health providers, 40.0% (CI: 34.4–46.0) of those with an NCD diagnosis were not taking prescribed medications, mainly blaming high costs. Those who sought care in the camp PHCC –or a private clinic– were much less likely to be taking medications as prescribed than those who sought care in hospital. It is likely that persons with more serious conditions sought care from hospital specialists, and their conditions may have been more advanced. This is a potential cause for concern as undertreatment or treatment interruptions risk development of complications. Impairment resulting can further limit function and increase dependency on other household members. As the economic situation of IDP households remains tenuous, identifying means to improve ready access to NCD medications at an affordable cost is critical.

The increasing financial crisis in the Kurdistan Region has resulted in the Ministry of Health reducing funding to BRHA camps. This is likely to affect the availability of health services. Delays and reduction in government employees’ salaries may weaken motivation among health providers. Doctors are likely to reduce the proportion of their time spent in public sector health facilities to further supplement their income through increased hours in private clinics [23]. Additional international support will be required to not only improve access, but also ensure the quality of care for persons with NCDs at the primary health care level, particularly if the health of Yazidis and other minority groups is to be maintained over an extended period of displacement.

## Conclusion

NCDs are common among displaced Yazidis and other minorities residing in BRHA camps. Although most displaced persons with an NCD diagnosis had seen a health provider for this condition in the 3 months preceding the survey, about 40% were not taking prescribed medications, mainly due to costs. Health status and access to care are likely to deteriorate in the coming months, as funding for the public sector declines and the economic situation of displaced households becomes even more tenuous. New strategies are needed to provide high quality NCD management at the primary health care level in the camps and ensure access to affordable medications.

## Abbreviations

BRHA: Board of Relief and Humanitarian Affairs
IDP: Internally Displaced Person
ISIS: Islamic State of Iraq and Syria
NCD: Non-Communicable Disease
PHCC: Primary Health Care Clinic
